# An experimental study to measure oil recovery factor by chemical agents and carbon dioxide after waterflooding

**DOI:** 10.1038/s41598-022-13639-7

**Published:** 2022-06-08

**Authors:** Guilin Yang, Yunyun Bai, Yuanjuan Song, Ahmed Sayed M. Metwally, Omar Mahmoud

**Affiliations:** 1 Department of Geology, Northwest University, Xian, 710069 Shaanxi China; 2 School of Chemistry and Chemical Engineering, Yulin University, Yulin, 719000 Shaanxi China; 3 No. 2 Oil Production Plant, Changqing Oilfield Company, PetroChina, Yulin, 719000 Shaanxi China; 4 Department of Mathematics, College of Science, King Saud University, Riyadh, 11451 Saudi Arabia; 5 Petroleum Engineering Department, Faculty of Engineering and Technology, Future University in Egypt, New Cairo, 11845 Egypt; 6 Department of applied sciences, University of Mon, Mon, Belgium

**Keywords:** Carbon capture and storage, Energy storage

## Abstract

Development of tight formations would be one of the main priority for petroleum industries due to the enormous demand to the fossil fuels in various industries. In this paper, we provided a set of experiments on the generated foams by carbon dioxide (CO_2_) and nitrogen (N_2_), cyclic CO_2_ injection, water alternating gas injection (WAG), active carbonated water injection (coupling surfactant effects and carbonated water (CW)), and introducing the impact of active carbonated water alternating gas injection (combination of WAG and CW injection) after waterflooding. Carbon dioxide is more feasible than nitrogen, it can be mobilize more in the pore throats and provided higher oil recovery factor. Generated foam with CO_2_ has increased oil recovery factor about 32% while it’s about 28% for generated foam by N_2_. Moreover, according to the results of this study, the maximum oil recovery factor for active carbonated water alternating gas injection, active carbonated water injection, and water alternating gas injection measured 74%, 65%, and 48% respectively.

## Introduction

Increasing demand to energy which has been supplied by fossil fuels provide a straight forward way to invest more on tight hydrocarbon reservoirs regarding the less productivity from conventional reservoirs. Therefore, development of tight formations would be one of the main criteria for petroleum industries. Waterflooding can be widely used for oil recovery purposes, however, in tight reservoirs is not beneficial enough as the oil production will decrease sharply after the water breakthrough. Thereby, this method can’t be used as the main enhanced oil recovery techniques and additional chemical or thermal methods would be more demanding to increase the oil production after waterflooding. Wide applications of foams in enhanced oil recovery (EOR) techniques which can be applied as the blocking agent especially for high permeable pores to conduct the more feasible sweep efficiency for oil in low permeable pores is one of the main features of implementation of this chemical agent. Foams injection is one of the most appropriate chemical enhanced oil recovery methods especially in tight oil reservoirs^1–3^. Hu et al. (2020) experimentally investigated the coupling effects of foams and various saline brines on the enhanced oil recovery factor. It was observed that coupling injection of foams and KCl would be the optimum injectivity scenario. The reason for this oil recovery enhancement corresponds to the monovalent property of K^+^ on the wettability alteration. Another findings of this study related to its lowest interfacial tension which would be a good feature for oil recovery purposes^4^. Among chemically enhanced oil recovery techniques, surfactant has been always been an interesting topic among scientists and petroleum companies. It is corresponded to the useful characteristics of surfactants to alter the wettability and reduce the interfacial tension which has caused to increase the oil recovery factor in tight reservoirs. For example, Nguyen et al.^5^ observed that wettability alteration was the main phenomenon on the oil recovery increase in tight oil reservoirs.

Injection of carbon dioxide in tight oil reservoirs would be of important as it can cause oil swelling and subsequent oil viscosity reduction. It is led to increase oil recovery factor^6,7^. When the carbon dioxide (henceforth; CO_2_) has been injected under miscible condition, there is no interfacial tension between oil and CO_2_ that can be a useful method for enhanced oil recovery in tight reservoirs. On the other hand, cyclic injection of carbon dioxide in tight reservoirs would be a prominent technique to increase oil production as the CO_2_ has the sufficient time to soak the core samples and dissolve in oil. This method was used by previous scientists and the results of this study would be consistent with the previous literature^8^.

When CO_2_ dissolved in formation brine (called carbonated water injection (CWI)) by the purpose of increasing the sweep efficiency, CO_2_ can transfer to the oil phase from formation brine. In this situation, CO_2_ can cause to decrease the oil viscosity and swelling^9^. According to the observation of some researchers, addition of surfactants to the carbonated water can be a crucial role on the wettability alteration which provide better efficiencies in oil recovery scenarios. It is called active carbonated water injection which couple the considerable effect of surfactants and carbonated water injection to reduce the oil viscosity. Water alternating gas injection is one of the effective EOR methods as it can inject both phases of water and gas in turn^10,11^. However, Yu et al. (2019) observed that carbonated water injection in tight reservoirs in provided better results than water alternating gas (WAG) injection^12^. Here, we compare the active carbonated water injection with WAG injection scenario to justify that ACWAGI (active carbonated water alternating gas injection) would provide better results than WAG injection.

In this paper, we provide a set of experiments on the applications of foams (generated by CO_2_ and N_2_), cyclic carbon dioxide (focused on the number of cycles due to more soaking time), water alternating gas injection, active carbonated water injection (coupling surfactant effects and carbonated water), and introducing the impact of active carbonated water alternating gas injection (combination of WAG and CW injection) after waterflooding. In second section, the materials and experimental procedures has been explained in more detail and then in third section, the experimental results of this paper were separately illustrated for each scenario. Finally, the most notable features of this study is written in summary.

## Methodology

### Materials

#### Core samples

In order to provide reliable results, we brought 10 core samples from one of the tight reservoirs located in Daqing field (one of the unconventional oilfields in China). More information about the sedimentology and lithology of this oilfield can be found in^13^. The properties of core samples are explained in Table 1. The length of all the core samples are approximately 2 $\pm (\varepsilon =0.01)$ with the diameter of 2 ft.

**Table 1:** Rock characteristics of core sample properties.

No	Porosity (%)	Permeability (mD)
1	4.62	0.056
2	4.86	0.094
3	4.25	0.015
4	4.67	0.065
5	5.07	0.131
6	4.92	0.115
7	5.28	0.143
8	5.16	0.14
9	5.65	0.156
10	4.38	0.022

#### Fluids

##### Crude oil composition and properties

The crude oil that is used in this experiment is mainly contained heavy hydrocarbons (C^7+^) which is measured in the room temperature of 25 $^\circ{\rm C}$ and atmospheric pressure. The content of C^7+^ is 76.08 mol%, while lighter hydrocarbons only consisted less than 25% of the crude oil (23.92 mol %) which is indicated that crude oil is heavy and needed secondary and tertiary enhanced oil recovery techniques. The density and the viscosity of the crude oil were measured in the temperature of 120 $^\circ{\rm C}$ (reservoir temperature) 0.735 g/cm^3^ and 7.41 mPa.s respectively. Due to the higher viscosity of crude oil than live oil (1.52 mPa.s), the heavy oil can’t be swept efficiency.

##### Synthetic brine

The synthetic which was used in this experiment has the total salinity of 5878.6 mg/L. The carbonated water is prepared with the 0.35*10^–3^ mol/cm^3^ concentration of purified carbon dioxide (99.9%) under test situations.

##### Surfactant

Cetyltrimethylammonium bromide (henceforth; CTAB) is a quaternary ammonium surfactant which was used to generate foaming agents. It was widely used in enhanced oil recovery methods in previous literature^14^. The surfactant solution was prepared in 30,000 ppm and Critical micelle concentration (CMC) was obtained as 0.12 wt%. Therefore, to generate the foaming agent and prevent foam films, the surfactant concentration was kept at 0.1 wt%.

### Experiments and procedures

#### Experimental apparatus

**Figure 1:**
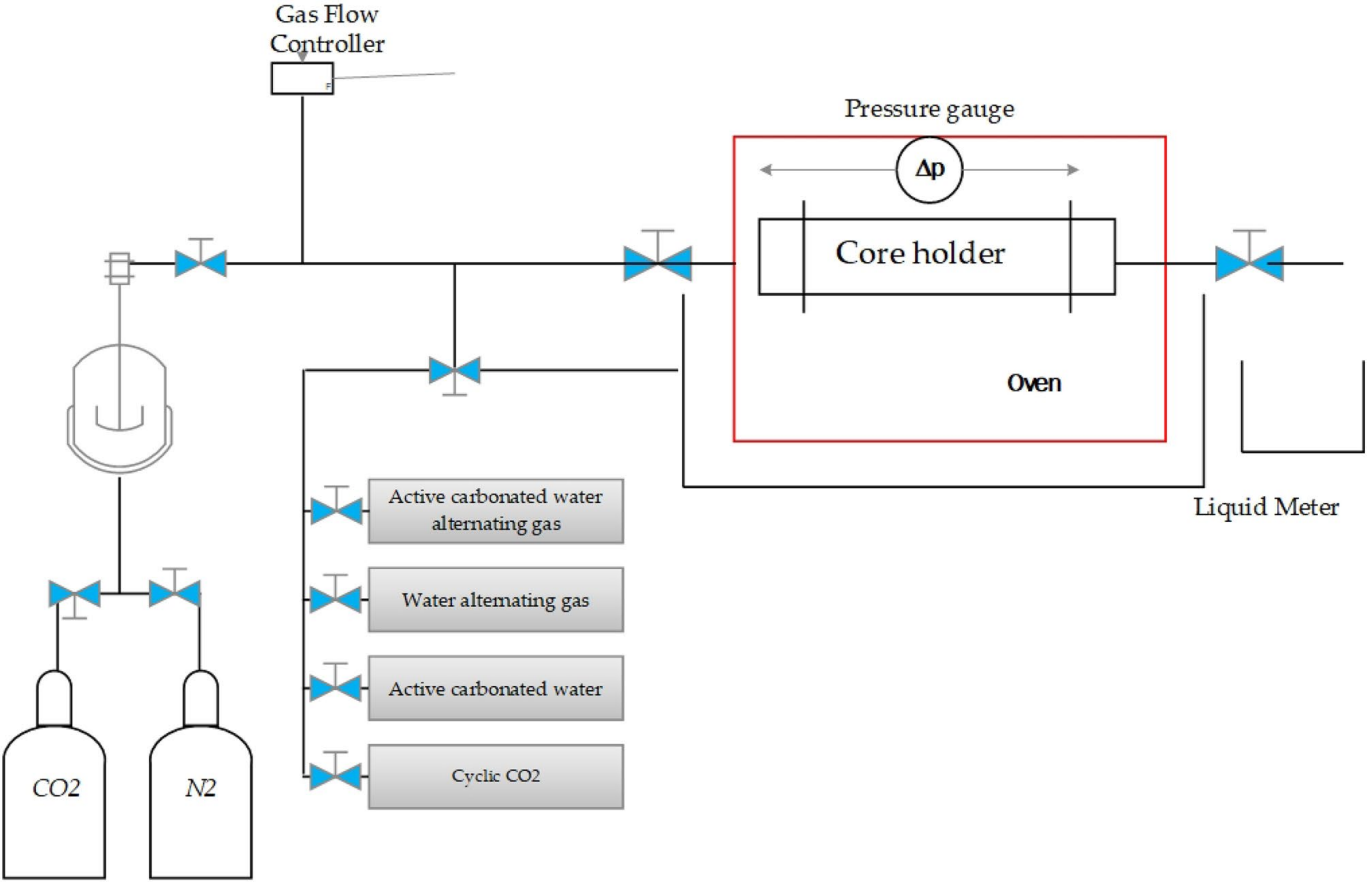
Experimental apparatus for coreflooding.

Figure 1 shows the experimental coreflooding apparatus schematically. The chemical agents are stored in a separate vessel and connected to the core holder (the place where tight core samples were fixed during the experiment) by some valves which was manually opened and closed. The core holder system was put in an oven with the defined reservoir temperature of 120 $^\circ{\rm C}$. The backpressure valve was used to control the pressure drop which was allocated to not allow the carbon dioxide liberation during the carbonated water injection. The liquid meter was defined in the system to measure the recovered flow which can be used to calculate oil recovery factor and water saturation.

#### Coreflooding procedure

The following procedure was done sequentially;

- To measure tight core samples’ permeability, all the core samples were died and then weighed. Next, by the high purified nitrogen gas, the permeabilities of the tight core samples were measured (See Table 1).
- The tight core samples were put in the core holder system horizontally in the defined reservoir temperature of 120 $^\circ{\rm C}$ and 25 MPa (confining pressure).
- The synthesized brine (to saturate core samples) and oil was injected sequentially with the flow rate of 0.02 cm^3^/min to measure the oil and water saturation.
- To calculate the oil recovery factor during the waterflooding, 2 PV of synthesized water was injected.
- Foams injection (generated by N_2_ and CO_2_), cyclic carbon dioxide injection, carbonated water injection, and active carbonated water injection scenarios were done separately at various pore volumes to measure oil recovery factor (Core samples 1 and 2).
- Finally, 1 PV of synthesized water was injected into the core samples to make sure that there is no remained oil in the core samples.
- Cyclic injection of carbon dioxide as a miscible phase for 1, 2, 5, 10, and 15 cycles has been performed (Core samples 3–7).
- Water alternating gas injection with the ratio of ½ (1 for water and 2 for gas) was performed after waterflooding on core sample 8.
- Carbonated water injection and active carbonated water alternating gas injection were done by core samples 9 and 10.

## Results and discussion

### Enhanced oil recovery

#### Waterflooding

**Figure 2:**
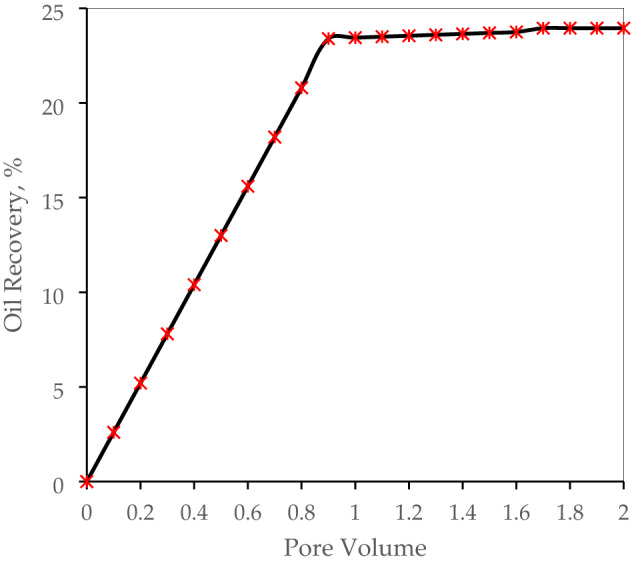
Oil recovery measurement for waterflooding.

First, we focused on the waterflooding scenario as enhanced oil recovery methods to compare with other recovery techniques. It is observed that the maximum value of oil recovery factor by water flooding is approximately 24% after 2 PV of water injection. At this point, there is no progress on the oil recovery factor and we implemented other injectivity scenarios (see Fig. 2).

#### Foams injection

**Figure 3:**
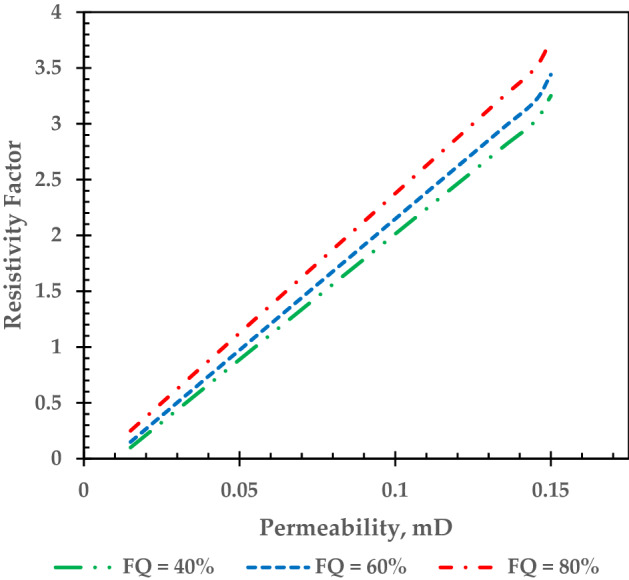
Resistivity factor measurement for different foam quality.

According to our experiments, it was observed that waterflooding can only be applied until 2 PV injection as it was reached a plateau on the oil recovery factor and it’s not significant change on the oil production. Then, we shifted to other enhanced oil recovery (EOR) methods after waterflooding to observe the value of increasing oil recovery factor. Due to the enormous applications of foams in EOR techniques which can be applied as the blocking agent especially for high permeable pores to conduct the more feasible sweep efficiency for oil in low permeable pores, in this part, we injected to types of foams after waterflooding. One was generated by the carbon dioxide and the second one generated by nitrogen gas. As resistivity factor is one of essential issues on the selection of foaming agent to have higher efficiency in EOR methods. Therefore, we select foam qualities of 40%, 60%, and 80% to measure resistivity factor for various permeabilities. By the increase of permeability, foam resistivity factor has been increased and foam quality of 80% has the highest value which indicated the more efficient sweep efficiency by the foaming agent. Thereby, foam quality of 80% was selected for this experiments (see Fig. 3).

**Figure 4:**
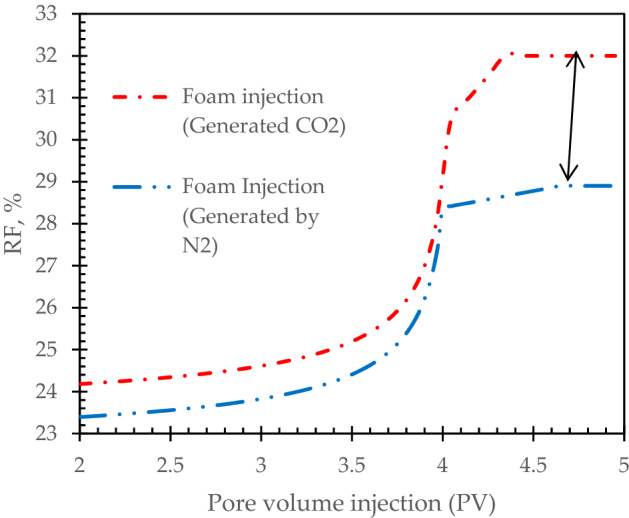
Comparison of foams injection with CO_2_ and N_2_.

Here, we used CO_2_ and N_2_ to generate foams. Then, we started the experiments after 2 PV of waterflooding to see the maximum value of oil recovery increase. In the first pore volumes of foams injection, oil recovery factor has increased slightly through the core samples indicated that foams were moving through the higher permeable areas to block them. Then, oil recovery has increased rapidly due to the blocking of high permeable areas and remained oils in low permeable areas displaced more feasible. It is corresponded to the foams efficiency to block the high permeable pores and pore throats. This point (PV = 4) is the commencement of oil recovery enhancement by generated foams. Carbon dioxide is more feasible than nitrogen, it can be mobilize more in the pore throats and provided higher oil recovery factor. Generated foam with CO_2_ has increased oil recovery factor about 32% while it’s about 28% for generated foam by N_2_ (see Fig. 4). It is corresponded to the weak solubility of N_2_ in crude oil due to the weak mass transfer capacity. It is caused to decrease the oil recovery factor which was discussed in previous literature^15^.

**Figure 5:**
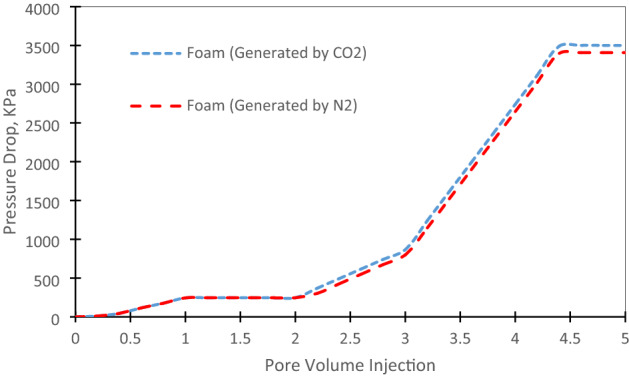
Pressure profile for foams injection.

Pressure profile for foam injection with CO_2_ and N_2_ is shown in Fig. 5. As it can be seen, before starting the foams injection, the pressure drop has not changed enough and it was observed about 246 kPa when the highest oil recovery was achieved by water flooding. Then, it has slowly increased as the foaming agent should block the high permeable throats first and then push oil from low permeable areas. It has its maximum pressure drop at 4.4 PV and after that it was reached a plateau which indicated that there is no increase on the oil recovery factor.

#### *Cyclic CO*_*2*_* injection*

**Figure 6:**
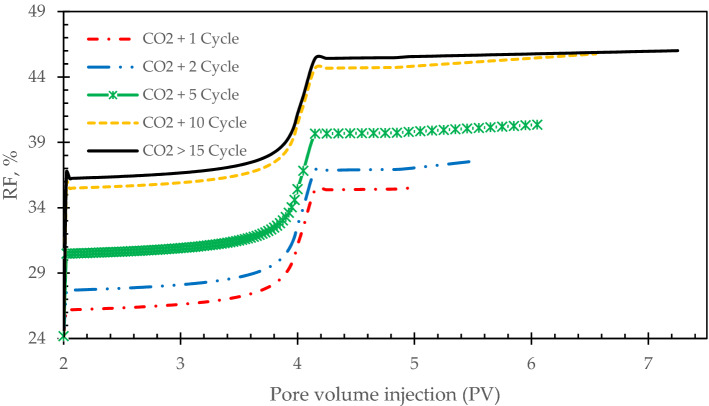
Impact of cycles’ numbers on the oil recovery factor.

In this part, we experimentally investigated on the cyclic injection of carbon dioxide as a miscible phase for 1, 2, 5, 10, and 15 cycles. Performing carbon dioxide experiments after 2 PV of waterflooding for different cycles would allow core samples to soak properly and then oil can be produced from the end of the core holder. This process is called huff-n-puff which was previously studied in literature^16–19^. Carbon dioxide injection is enhanced oil recovery slightly as it needs be solved as a miscible phase in the crude oil to reduce the oil viscosity. The minimum miscible pressure is recorded 8.14 MPa. This is corresponded to low oil recovery factor in the first stages of pore volume injection. Cyclic carbon dioxide injection can help to reach the minimum miscible pressure in a short time and this is why it can be trusted for enhanced oil recovery processes. By the increase of cycle’s number, oil recovery has improved, however, it is observed that this increase in the number of cycles would have a limit and after 15 cycles, there is no significant changes in the oil recovery factor. It would be an important guide for petroleum companies to test and distinguish the proper number of cycles as it needs high expenditures too in field applications (see Fig. 6).

**Figure 7:**
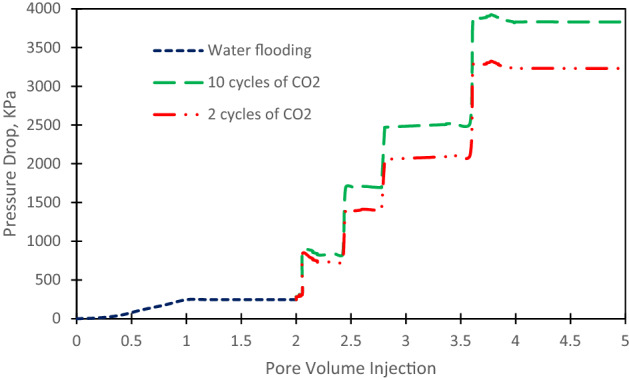
Pressure profile in cyclic CO2 injection.

Pressure profile for Cyclic CO_2_ injection is shown in Fig. 7. As it can be seen, before starting the Cyclic CO_2_ injection, the pressure drop has not changed enough and it was observed about 246 kPa when the highest oil recovery was achieved by water flooding. Then, it has slowly increased as the Cyclic CO_2_ injection and then dispalce oil from low permeable areas. It has its maximum pressure drop at 4 PV and after that it was reached a plateau which indicated that there is no increase on the oil recovery factor. By the increase of cycles number, pressure drop has been increased, which in cycles more than 10, the pressure drop is not changed a lot.

#### Surfactant- based methods

**Figure 8:**
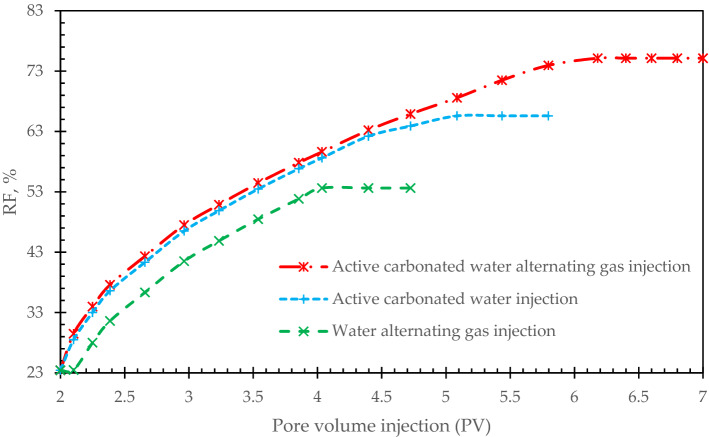
Oil recovery factor for various surfactant-based methods.

In this part, we experimentally investigated the effect of active carbonated water injection (which was made by CTAB as surfactant agent) and active carbonated water alternating gas injection (a mixture of water alternating gas injection and carbonated water which is made by CTAB. The rock-injected fluid interactions was one of the main features of carbonated water injection that caused to wettability alteration^20–22^. Therefore, it is necessary to perform each test individually and compare the results of each test together to select the optimum injectivity scenario. Figure 8 shows combination of water alternating gas and active carbonated water injection would be a more appropriate scenario on the oil recovery enhancement. Active carbonated water alternating gas injection has witnesses the highest oil recovery of 74%. The second highest oil recovery factor is allocated for active carbonated water injection. It is reported of 65%, while water alternating gas injection has improved the oil recovery factor to 48%.

**Figure 9:**
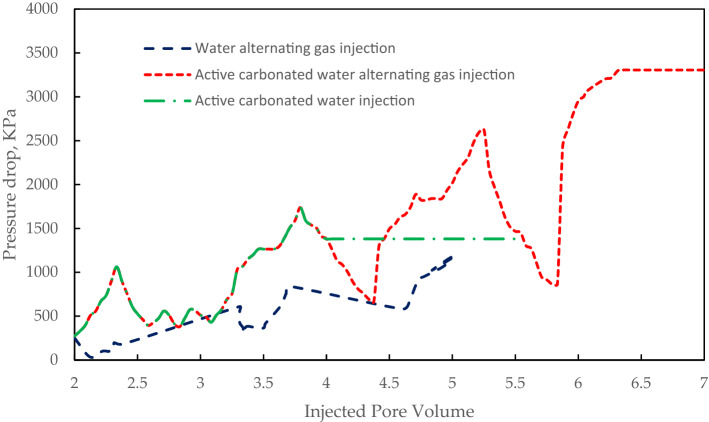
Pressure profile for three injectivity scenarios.

Pressure profile for these injectivity scenarios is shown in Fig. 9.

## Conclusions

- Active carbonated water alternating gas injection has the highest oil recovery factor of 74%. The second highest oil recovery factor is allocated for active carbonated water injection. It is reported of 65%.
- By the increase of cycle’s number, oil recovery has enhanced, however, it is observed that this increase in the number of cycles would have a limit and after 15 cycles, it’s not economically beneficial to continue the process.
- In the first pore volumes of foams injection, oil recovery factor has increased slightly through the core samples. It is corresponded to the foams efficiency to block the high permeable pores and pore throats.
- Carbon dioxide is more feasible than nitrogen, it can be mobilize more in the pore throats and provided higher oil recovery factor.
- Generated foam with CO_2_ has increased oil recovery factor about 32% while it’s about 28% for generated foam by N_2_. It should be noted that the observed data were normalized to provide more feasible results.

## Data Availability

All the data were used in this paper are included in the text.
